# Linking music streaming platform advertisements with a digital mental health assessment and interventions

**DOI:** 10.3389/fdgth.2022.964251

**Published:** 2022-11-01

**Authors:** Luke Balcombe, Diego De Leo

**Affiliations:** Australian Institute for Suicide Research and Prevention, School of Applied Psychology, Griffith University, Brisbane, QLD, Australia

**Keywords:** Mental health care, music-based interventions, engagement, linking, digital mental health assessment and interventions

## Abstract

Accessibility issues and low rates of help-seeking hinder engagement with mental health resources and treatment. Pragmatic, (cost-)effective solutions are required to increase engagement with efficacious digital mental health interventions (DMHIs) including for hard-to-reach individuals. As an example, music-based interventions have been positively used in health care to reduce stress, anxiety and depression through music medicine, music therapy and recreational use. Although, enhanced mental health awareness from music listening has yet to be converted into engagement with a DMH assessment (DMHA) and DMHIs. Therefore, a new study is proposed to place linked advertisements on Spotify, the most used music streaming platform. MindSpot's vetted DMHA is suitable to use as an example for linking unto because it measures depression, anxiety, general mental well-being problems and psychological distress in Australian adults and provides access to DMHIs. The primary aim is to provide a convenient, robust and scalable consumer pathway to reduce engagement barriers and maximize facilitation to a vetted DMHA and DMHIs. The proposed study is important because it addresses notorious help-seeking difficulties in the adult population (e.g., young people and men). It also expands outreach to the underserved and the unserved and streamlines the integration of digital solutions with mental health services.

## Introduction

Integrative engagement strategies are required to link Australians suffering with mental health problems to existing evidence-based digital mental health interventions (DMHIs). The 2020–21 National Study of Mental Health and Wellbeing reported that of the 21.4% (4.2 million Australians) with a mental health problem, 17% received support, of which only 9.7% received the help they require in terms of information and counselling (1). There is a dire need for responsible investments to reduce the compounding costs of mental illness and suicide (e.g., estimated direct economic costs at between AUD$43 billion and $70 billion, with an additional $151 billion due to the cost of disability and premature death in Australia) (2). The challenges may become more serious when considering suicide rates are likely to rise as the economic and mental health impact of COVID-19 develops over time (3). Out of the AUD$2.3 billion budget over four years for Australia's National Mental Health and Suicide Prevention Plan (4), the main expenditure items are treatment and suicide prevention. These main expenses have been evaluated as ineffective and may be futile in some cases given the limited treatment options available as well as the scarcity of mental health care resources particularly for treatment-resistant patients (5). A variety of barriers affect mental health help-seeking such as geographic isolation, stigma, attitudes and behaviors (6–8).

Digital mental health (DMH) services were accessed by 4.4% of Australian people aged 16–85 years (864,100 people) (1). Currently, DMHIs are sourced from self-initiated searches for online mental health information resources or through guidance from healthcare professionals. These tools consist of a course or module containing a text description of the topic, a self-rating questionnaire and psychoeducational resources in text or video. There is growing evidence for DMHIs mostly through Internet-delivered cognitive behavior therapy (ICBT) for anxiety and depression (9–12), and to a lesser extent for obsessive-compulsive disorder (13) and insomnia (14), in addition to treatments focused on online self-help mindfulness (15). A recent Australian-focused scoping review found 52 evidence-based DMHIs for a range of indications: depression (*n* = 9, 17%); anxiety (*n* = 15, 29%); general mental well-being (*n* = 13, 25%); multiple issues (*n* = 13, 25%); and distress in the form of suicidal ideation (*n* = 2, 4%) (16). Although many DMHIs are of a good quality and circumvent many of the barriers to help-seeking (17–20), uptake remains low, particularly in priority populations such as males, indigenous Australians, and those of low socio-economic situations (21–23). Uptake is low because of the overwhelming choice of apps (24) and uncertainty among both practitioners and consumers in which ones are efficacious (25).

Improved collaboration between DMH services and academic teams is required to support a better understanding of user experience (UX) and engagement (26). A meta-analysis found that the more that users engage with DMHIs the greater the improvements in mental health symptoms (27). This finding points to the importance of providing access to strategies for those individuals who are less motivated to engage or experience more barriers to engagement.

In summary, there is a significant socio-economic impact from mental illness and suicide which is exacerbated by the barriers to help-seeking and the limitations of traditional mental health care resources. Although digital solutions offer the opportunity to reduce the strain on mental health care resources and increase help-seeking among nearly all populations, clinicians and consumers need to be careful in selecting a DMHI to recommend and use. Subsequently, providing effective DMHIs should remain a major research focus. However, another primary gap is the need to better understand engagement especially for those who have experienced barriers or are less inclined to self-initiate the DMHI process. It is therefore proposed to explore music-based interventions used in health care as a possible way to increase engagement with reliable digital solutions.

## Music-based interventions used in health care and the potential for digital approaches

Music-based interventions (i.e., music medicine, music therapy, and other music-based interventions) are used globally in mental health care settings and private practice, as well as in schools and geriatric facilities. Music medicine involves listening to pre-recorded music for health promotion provided by health care professionals. Music listening has positive physiological and psychological effects (e.g., reducing stress, anxiety and depression) (28). Music therapy is practiced for addressing clients' physical, emotional, cognitive, and social needs (29) through music listening, writing songs, singing and/or playing an instrument. A review of five randomized trials that investigated music therapy for depression found acceptability and positive mood changes (30). Music therapy applications have been progressively used globally, for example in clinical treatment of children and adolescents (31) with evidence for reducing depression (32) and internalizing symptoms (33). Other music-based interventions consist of recreational goals as an alternative to health promotion and are not necessarily provided by health care professionals (e.g., musicians may be a source).

**Figure 1:**
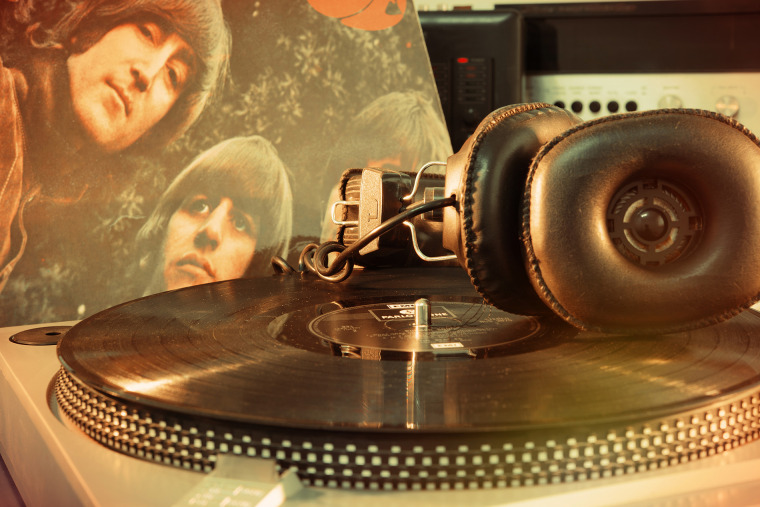
A record by The Beatles (Rubber Soul, released in 1965). De Bernado, V. (2013). Vintage Beatles album; stock photo ID:472081965. Sourced from: https://www.istockphoto.com/photo/vintage-beatles-album-gm472081965-31665986 (viewed on 20 May 2022).

Music streaming has provided a noticeable change in accessibility to music. Music streaming platforms (e.g., Spotify, Apple Music, Pandora Radio and SoundCloud) accessed through desktops, tablets or smartphones are the most used mode of music listening. Compact discs (CDs) and records (see Figure 1) are still used but to a lesser extent. There are more than 80 million music tracks available on Spotify and more than 265 million music tracks on SoundCloud. As of June 2022, there were nearly 524 million music streaming subscribers throughout the world (34). Spotify is globally the most used platform with 422 million unique users. Therefore, 234 million people (55.5%) use the platform for free (i.e., with advertisements) and 188 million are premium subscribers (i.e., advertisement-free access) (35). The digital delivery of pre-recorded music has seen a significant increase since 2017, when it was last recommended to use technology to translate positive findings of music listening interventions research into pragmatic (36), cost-effective (37) health applications. This has yet to be realized although there are signs the music streaming industry is receptive to such applications. For example, Spotify's “tune into yourself” text and audio advertisement to mark World Mental Health Day as well as the “Let's Talk About Mental Health” podcast which promotes normalizing the conversation about mental health and mental illness to reduce stigma.

**Figure 2:**
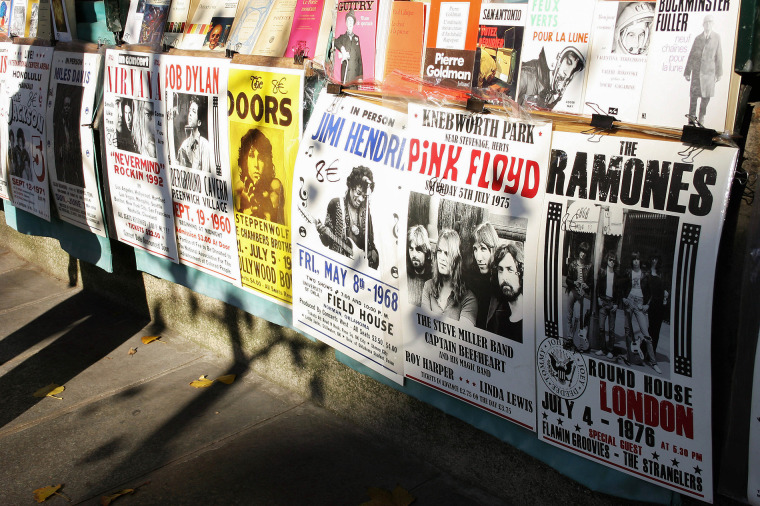
Replica concert posters including popular musicians of the 1960s-1990s. Aydin, D. (2015). Concert Posters from 60's and 70's; stock photo ID:480624340. Sourced from: https://www.istockphoto.com/photo/concert-posters-from-60s-and-70s-gm480624340-69030717 (20 May 2022).

The change in patterns of music listening means that including digital delivery in music-based interventions is recommended to keep apace. As a start, the definition of music-based interventions should be enhanced to include digital approaches. For example, video conferencing may have been applied more commonly in music therapy during COVID-19. Music medicine may benefit from the digital listening of pre-recorded music. Recreational music listening experiences could be better harnessed to maximize mental health promotion inclusive of digital delivery. Since the 1960s, electronics expanded music delivery through live concerts (see Figure 2), events, and karaoke. Pre-recorded music listening experiences have been linked to technological advances through the mediums of radio, stereo, headphones and more recently music streaming and wireless earphones.

A feature of digital music listening experiences is that song lyrics are readily available. For example, it is possible to follow song lyrics in real-time on the Spotify app. The potential to integrate such functions with mental health support is apparently unexplored in the literature. It may be useful for therapists to incorporate music streaming platforms in understanding their clients' musical interests for assessment and therapy purposes. For example, a Spotify playlist of 20 songs about men's mental health was made to demonstrate songs by men that apparently include content about addiction, anxiety, depression, bipolar disorder, loneliness, schizophrenia, psychache and/or suicidality. The therapist may use such a playlist and ask the client to explain the significance of the songs, interpret them and to discuss emotions. There is a potential for the lyrical content and the context of songs to be misunderstood. For example, Australia's unofficial national hymn “Waltzing Matilda” - about a swagman who died by suicide after being caught stealing a sheep at a waterhole - is thought to represent the Australian spirit (38). A literal interpretation of the lyrics may consider it to be a glorification of suicide. However, the song's context commonly represents the enculturation of rugged individualism - an anti-hero rebelling against the constraints of colonialism (39).

The literature suggested various ways in which music may be used to improve mental health: as a medicine or therapy; as a feature of mental health support; and to increase awareness of mental health. A relatively small number of studies on music-based interventions to-date found positive mental health outcomes, for example in reducing stress, anxiety and depression. Music medicine and music therapy could be revived by exploring digital solutions. In addition, the recreational use of pre-recorded music and podcasts has shown promise in mental health promotion on Spotify. However, enhanced mental health awareness from music streaming has yet to be effectively harnessed in research. This gap may be filled through promoting a vetted DMH platform on Spotify to increase engagement with a DMH assessment (DMHA) and DMHIs.

## Linking Spotify advertisements to a DMHA and DMHIs

The remarkable increase in the use of music streaming platforms and DMHIs has not yet been translated into research that combines these approaches. The nearest concept is online gatekeeping which involves placing advertisements on web search pages to promote mental health consultation services. For example, studies of online gatekeeping for suicide prevention found that online gatekeeping may be feasible (40) and could be more successful if targeted approaches are provided with indications of individual problems (e.g., daily life, financial or work) (41). The paucity of research of online gatekeeping means that there is an unrealized opportunity to explore beyond what might be “traditionally” considered to be connecting consumers to mental health support.

DMHIs have the potential to reach nearly all Australians, yet they don't because of uncertainty over their efficacy and a lack of innovative outreach. This justifies the proposal to place mental health promotion advertisements on Spotify which link to a vetted DMHA. The Australian context is suitable for introducing research on linking Spotify with DMH services because of the rapidly increasing use of these digital solutions. As of June 2020, 12.7 million Australians (61% of the overall population) use music streaming services in an average four weeks; 8 million of which use Spotify which is the leading platform ahead of You Tube with 5.5 million users (42). As noted in the Introduction, 864,100 Australians (4.4% of people aged 16–85) accessed DMH technologies in 2020–21(1). Although there are no comparative statistics available from the previous National Survey of Mental Health and Wellbeing in 2007, it can reasonably be derived that the use of DMH technologies has significantly increased in recent years (16) as part of early intervention strategies for young people (43) and in addressing help-seeking and service gap issues for adults (44).

MindSpot is proposed as a suitable example of a DMH platform because it provides a clinically validated, personalized, free, online self-guided DMHA for adult Australians. MindSpot's DMHA administers the Patient Health Questionnaire-9 (PHQ-9), Generalized Anxiety Disorder 7-Item Scale (GAD-7), and Kessler Psychological Distress 10-Item Plus Scale (K-10+) as standardized measures for depression, anxiety, general mental well-being problems and psychological distress (9). The DMHA text description explains that it takes 20–30 min to complete and is about the impact of the users' physical and emotional symptoms.

The proposed study aims to:

• Assess a scalable model of delivery for effectively linking Spotify mental health promotion advertisements with MindSpot's free, online self-guided assessment for Australian adults with symptoms of anxiety, depression, general well-being problems and/or distress.
• Conduct a mixed methods online survey of the perceptions of a sample of Australian adults in terms of the usability and quality of linking Spotify to MindSpot's online self-guided assessment.
• Develop recommendations on how to maximize user engagement with a DMHA and treatment.

## Approach

### Participants

The number of people who engage in the linking phase of the study (i.e., those recruited to MindSpot from Spotify advertisements) will determine the number of participants. It is aimed for 500,000 Spotify advertisements to be served although the click-through-rate (CTR) is unknown. There may be 300–10,000 MindSpot users based on the range of Spotify's previous CTRs although the higher end may be possible as the advertisements are planned to coincide with Spotify's World Mental Health Day campaign plus the DMHA is free. The user feedback survey sample size will require at least 120 participants for regression analysis (i.e., to describe the impact of overall findings).

### Measures

The number and metrics of people who were (1) reached by and (2) engaged with the MindSpot linking advertisement on Spotify will be measured from Spotify's Ad Studio dashboard analytics.

Mixed methods research is proposed for measuring user feedback combining the strengths of both qualitative and quantitative approaches (45) in survey research.

There are two components proposed for user feedback – usability and quality. Usability refers to how well a user can use linking and the DMHA to achieve psychological screening of their mental health. Quality refers to the user experience with the linking process to the DMHA e.g., how well it worked, how the user expected it to work, how they feel about using it, and how they feel about Spotify and MindSpot.

Data on the usability and quality of linking to the DMHA will be derived from Spotify's Ad Studio dashboard analytics and an online user feedback survey.

Data on the usability of linking will include advertising outreach attempts to users as well as the number of users who clicked on or touched the advertisement link (represented by the CTR). Task accuracy and task efficiency data can be obtained from MindSpot's digital platform which records user completion rates and the time taken to complete the DMHA. User satisfaction with the linking procedure will be derived from a user feedback survey with questions scored with a Likert scale and open-ended questions on users' perceptions of the linking procedure and the DMHA e.g., whether it is effective, efficient, engaging, error tolerant, and easy to use.

Data on the quality of linking will be derived through a user feedback survey that will be created based on Borghouts et al.'s (18) systematic review of the constructs influencing DMHI user engagement, grouped as constructs related to the user, the program offered by the intervention, and the technology and (implementation) environment. Out of 16 grouped constructs, the following 8 constructs will be investigated in the user feedback survey: demographic variables; mental health status; mental health and technology experience and skills; perceived fit; perceived usefulness; technology factors; privacy and confidentiality; and implementation.

### Procedure

**Figure 3:**
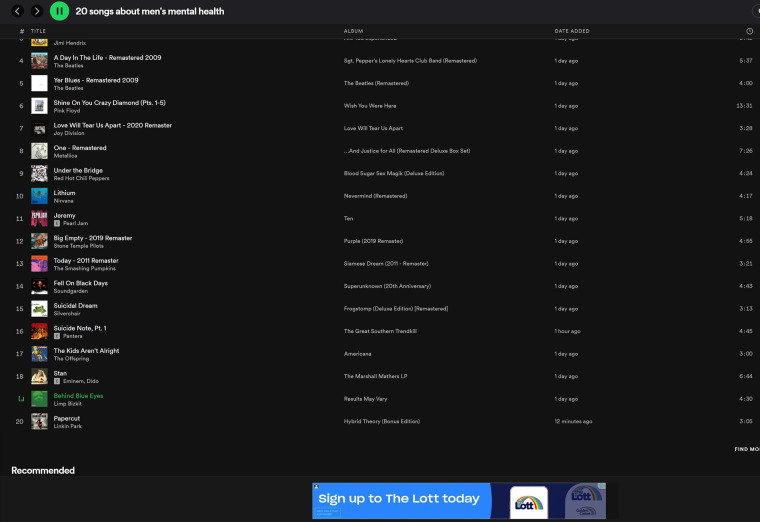
Spotify playlist with a branded image advertisement. Balcombe, L. (2022). Spotify playlist: “20 songs about men's mental health”. Sourced from: https://open.spotify.com/playlist/7yvyppDtEBwf6BDvnTwDNK?si=d38a55a2217f41aa (viewed on 20 May 2022).

**Figure 4:**
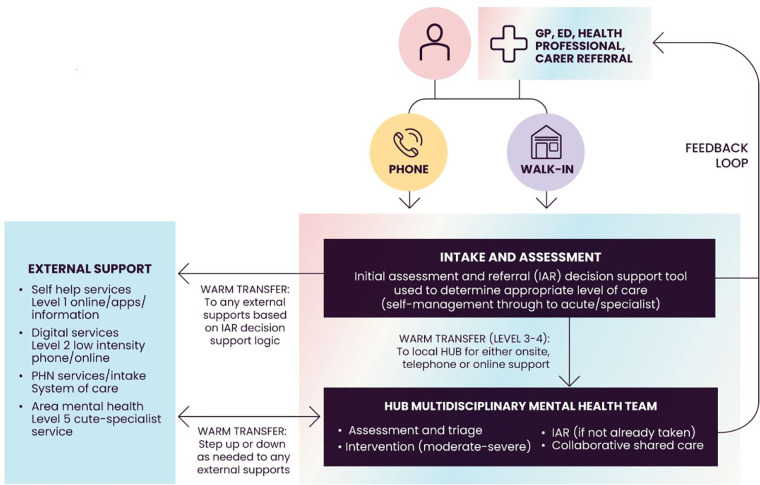
Head to Health access procedures for health care providers. Australian Department of Health (2019). National Initial Assessment and Referral for Mental Healthcare Guidance. Sourced from: https://headtohealthvic.org.au/for-health-care-providers/ (viewed on 26 August 2022).

1. Ethics – the project will undergo an ethical review and gain ethical clearance before any human research commences.
2. Advertising campaign - Spotify Australia's customized Ad Studio solutions will be applied including a branded image (see Figure 3) with audio.
3. Linking to MindSpot – the user is linked to the DMHA webpage which includes a text description of key details, assessment steps and why to complete an assessment. If the DMHA is completed, an automated summary of assessment results is provided in addition to recommendations for the next steps to consider. At this stage, the user may be able to follow MindSpot's online treatment programs. However, there are limited places available so users may alternatively be signposted to consult a GP or other healthcare professional who may organize a referral to Head to Health's intake and assessment service (see Figure 4) which has the capacity to serve the Australian population.
4. Link to User Feedback Survey – an invitation to the survey will appear after the user leaves the DMHA. If the user proceeds, an information and consent form is presented for this part of the study.

### Data analysis

A descriptive analytical dashboard with statistics and graphs will be provided for a comprehensive overview of the engagement metrics on linking Spotify advertisements with MindSpot's DMHA (e.g., the CTR, how many linked users were identified, and time spent on the DMHA, etc.).

The user feedback data will be analyzed through descriptive techniques. Statistics and regression analysis will describe the impact of overall findings for quantitative survey questions on demographic variables, mental health status, mental health and technology experience and skills, as well as user satisfaction. The synthesis of open-ended survey questions through qualitative analysis will provide valuable insights into users' perspective. An inductive thematic analysis (46) through NVivo will be used to identify common themes. Codes will be created based on what emerges from the data on perceived fit, perceived usefulness, technology factors, privacy and confidentiality, and implementation.

## Ethics

There is a potential for music-based interventions to lead to distress. For example, the existence of the country music subculture is related to increased risk for suicide. A study found the greater the airtime devoted to country music in American metropolitan areas, the greater the white suicide rate (47). However, music listening is not expected to cause suicide. A literature search of the suicidal impact of music found no other evidence in other music genres. For example, experiments with college students on the impact of suicidal-themed rock videos and music found those exposed to suicidal content were more inclined to have suicide-related ideation but there was no association with increased suicide risk (48). It is recommended that music medicine and music therapy users engage with academically and clinically trained professionals for tailoring appropriate music experiences and mental health support.

The proposal to use digital solutions should consider how to include people who do not have access to the internet, computers or devices. The scoping and development of Australia's National DMH Framework seeks to address findings that Aboriginal and Torres Strait Islander people, older Australians, people from low socio-economic backgrounds and rural and remote communities are the least digitally included groups in Australia (49). More efforts are needed to reduce the digital divide. Accessibility for those without functional and internet-connected personal computers or devices may be improved through the provision of digital resources at community centers, clinics or libraries.

It is possible that some people do not want the integration of mental health advertisements on music streaming platforms. It is recommended to provide simple yet effective mental health care messages as they can be interpreted differently than intended and may have a detrimental impact. An advertising period from late September to October 10 is proposed to coincide with Spotify's advertising campaign for World Mental Health Day. Co-design with mental health care and social marketing professionals is proposed to help the linking of advertisements with DMHIs to be as least obtrusive as possible. Likewise, it is important to integrate a human-centered design approach including user feedback to ensure equitable and meaningful engagement (50).

## Future research

Although it is proposed to engage with adults in the general population, the proposed study may be useful for informing targeted approaches in future research. For example, it may show the association between demographics and engagement levels with Spotify and the DMHA. It may also show how to get children and adolescents from priority populations to uptake DMH programs. This may involve working closely with GPs and schools in DMH promotion and introducing Spotify advertisement linking for engaging parents to involve their children in DMH programs as well as another linked Spotify ad campaign aimed at adolescents. Other research ideas might involve modification of the model of delivery. For example, it may become possible to integrate the DMHA with online GPs and a digital intake to Head to Health beyond the current phone and walk-in options. Furthermore, it is of interest to explore digital phenotyping (51) in future research to analyze Spotify user details, song subject type and use of songs to assist in identifying “at-risk” people in terms of mental illness or suicidality. This may involve applying machine learning algorithms to identify patterns of use in music streaming platforms. Determining appropriate users to target with Spotify's advertisement links for mental health promotion may be informed by pattern analysis of the types of songs played on the platform, the context of song lyrics and how they interact on the platform.

## Conclusions

The literature identified a lack of pragmatic, (cost-)effective solutions to address the problems of limited mental health care resources, low rates of help-seeking, as well as a lack of digital solutions in music-based interventions to divergently increase engagement with DMH services. The main aim of the proposed study is to create a convenient, robust and scalable mental health consumer pathway. It is a novel concept to seize the window of opportunity to engage in mental health while listeners of music streaming platforms are receptive and available with the time and headspace to follow the linking advertisement to a free DMHA. The proceeding steps are self-guided whereby the user explores the resources and treatment options before deciding whether to engage with DMHIs and/or in a face-to-face approach. The Australian Government supported MindSpot platform has a limited treatment capacity although users may alternatively access the national intake and assessment service, Head to Health, ordinarily through a GP. The new study has the capacity to make population-level mental health improvements and may serve as a streamlined example of a model of delivery for DMH outreach (e.g., to better serve young people and men).
